# Change in sprint cycling torque is not associated with change in isometric force following six weeks of sprint cycling and resistance training in strength‐trained novice cyclists

**DOI:** 10.1002/ejsc.12203

**Published:** 2024-10-16

**Authors:** Shannon Connolly, Peter Peeling, Martyn J. Binnie, Paul S. R. Goods, Wouter P. Timmerman, Toni Haddad, Chris R. Abbiss

**Affiliations:** ^1^ Centre for Human Performance School of Medical and Health Sciences Edith Cowan University Joondalup Western Australia Australia; ^2^ Western Australian Institute of Sport Mount Claremont Western Australia Australia; ^3^ School of Human Sciences (Exercise and Sport Science) The University of Western Australia Crawley Western Australia Australia; ^4^ Murdoch Applied Sports Science Laboratory Discipline of Exercise Science School of Allied Health Murdoch University Perth Western Australia Australia; ^5^ Centre for Healthy Ageing Health Futures Institute Murdoch University Perth Western Australia Australia; ^6^ South Australian Sports Institute Adelaide South Australia Australia

**Keywords:** performance, strength, testing, training

## Abstract

Strong relationships exist between sprint cycling torque and isometric mid‐thigh pull (IMTP) force production at one timepoint; however, the relationships between the changes in these measures following a training period are not well understood. Accordingly, this study examined the relationships in the changes of sprint cycling torque and IMTP force following six‐weeks of sprint cycling and resistance training performed by strength‐trained novice cyclists (*n* = 14). Cycling power, cadence, torque and IMTP force (Peak force [PF]/torque, average and peak rate of force/torque development [RFD/RTD], and RFD/RTD from 0 to 100 ms and 0–200 ms) were assessed before and after training. Training consisted of three resistance and three sprint cycling sessions per week. Training resulted in improvements in IMTP PF (13.1%) and RFD measures (23.7%–32.5%), cycling absolute (10.7%) and relative (10.5%) peak power, peak torque (11.7%) and RTD measures (27.9%–56.7%). Strong‐to‐very strong relationships were observed between cycling torque and IMTP force measures pre‐ (*r* = 0.57–0.84; *p* < 0.05) and post‐training (*r* = 0.63–0.87; *p* < 0.05), but no relationship (*p* > 0.05) existed between training‐induced changes in cycling torque and IMTP force. Divergent training‐induced changes in sprint cycling torque and IMTP force indicate that these measures assess distinct neuromuscular attributes. Training‐induced changes in IMTP force are not indicative of training‐induced changes in sprint cycling torque.

## Highlights

- Six weeks of resistance and sprint cycling training resulted in improvements in isometric mid‐thigh pull (IMTP) peak force (13.1%) and rate of force development measures (23.7%–32.5%), cycling absolute (10.7%) and relative (10.5%) peak power, peak torque (11.7%), and rate of torque development measures (27.9%–56.7%) in strength‐trained novice cyclists.
- No significant relationships were observed between the training‐induced changes in IMTP force measures and sprint cycling torque measures following training.
- No significant relationships were observed between the training‐induced changes in cycling peak power and changes in cycling torque, IMTP force, back squat and trap bar deadlift with three repetition maximum.

## 1 INTRODUCTION

The magnitude and rate at which a cyclist can apply force to the pedal to produce bicycle crank torque are important factors influencing mechanical power production and sprint cycling performance (Gardner et al., 2007; Watsford et al., 2010). Maximal torque (*T*_peak_) production is important in overcoming inertia to accelerate the bicycle, while the crank rate of torque development (RTD) is critical given the limited time available for torque production when cycling at moderate‐to‐high cadences. For instance, when pedalling at cadences of 110 revolutions per minute (rpm) or higher, the time available for muscular force production during the pedal downstroke can be less than the 300 ms needed to produce *T*_peak_ in important lower limb muscles such as the knee extensors during an explosive maximal voluntary isometric contraction protocol (Aagaard et al., 2002). Accordingly, a greater crank RTD early (i.e., first 100 ms) in the pedal downstroke will result in a steeper torque rise and thus greater impulse, which will increase mean crank power and bicycle speed.

Sprint cyclists typically dedicate a substantial amount of time to resistance training to increase muscle strength in order to increase RTD and peak power output (PPO) (Dorel, 2018; Kordi, Folland, Goodall, Menzies, et al., 2020). Accordingly, valid and sensitive measures to assess changes in parameters of neuromuscular function are of importance to our understanding of sprint cycling performance. Cyclist neuromuscular function is commonly assessed using cycle‐ and gym‐based measures (Connolly et al., 2023; Kordi, Folland, Goodall, Menzies, et al., 2020; Vercoe & McGuigan, 2018). On the bicycle, a torque‐velocity test allows the valid and reliable delineation of a cyclist's torque‐ or power‐cadence profile (Dorel, 2018; Wackwitz et al., 2021). While recent technological advancements in power meter technology allows for reliable cycling RTD measurement (Connolly et al., 2022), little is known about the training‐induced changes in this measure. In the gym, physical qualities such as maximal strength can be assessed using repetition maximum (RM) testing in movements such as squat or deadlift, whereas rate of force development (RFD) and PF are commonly assessed using isometric testing, such as the IMTP (Guppy et al., 2018).

Strength measurements (PF/torque, RFD/RTD and weight lifted) are typically evaluated across a range of gym and field assessments (e.g., IMTP, 3RM and sprint cycling). Knowledge of the relationship between training‐induced changes in these measures is important in understanding which neuromuscular factors are indicative of changes in cycling performance. Previous studies reported strong‐to‐very strong relationships between PF/torque (Vercoe & McGuigan, 2018, *r* = 0.89–0.93, *p* < 0.05; Connolly et al., 2023, *ρ* = 0.76, *p* < 0.01), early and late RFD/RTD (from 0 to 100 ms and 0–200 ms, respectively) and peak RFD/RTD (Connolly et al., 2023; r or *ρ* = 0.61–0.70; <0.05) produced during an IMTP and sprint cycling at one timepoint. However, an isolated relationship does not imply a causal relationship, and therefore, it is currently unclear whether the training‐induced changes in these qualities are related. Findings from our earlier work (Connolly et al., 2023) revealed differences in the underpinning neuromuscular function measures related to IMTP RFD and sprint cycling torque, indicating a possible diverging response to training. Indeed, previous research provides conflicting evidence around the training‐induced changes in neuromuscular function from strength assessments and cycling. For instance, Rønnestad et al. (2010) reported that an experimental group of 11 well‐trained cyclists increased isometric half‐squat PF by 21.2 ± 4.9% (*p* < 0.01) and cycling *P*_max_ by 9.4 ± 2.9% (*p* < 0.01) following a 12‐week intervention of endurance cycling and lower body heavy strength training. In contrast, Kordi, Folland, Goodall, Menzies, et al. (2020) observed a moderate relationship (*r* = 0.42–0.47; *p* < 0.05) between the training‐induced changes in knee extension late RTD (150–200 ms) and cycling *P*_max_ following a 6‐week isometric cycling specific strength intervention in 24 elite track sprint cyclists. Given this, future research is warranted to determine the relationship between training‐induced changes in strength assessment and cycling neuromuscular function.

To this end, the aims of the present study were to investigate: (i) the extent that sprint cycling torque and IMTP force measures change following training; and (ii) the relationship between training‐induced changes in sprint cycling torque, cycling power, IMTP force and 3RM measures. Based on our earlier findings (Connolly et al., 2023), it was hypothesised that the training‐induced changes in IMTP force and sprint cycling torque would not be related. Further, based on previous findings (Kordi, Folland, Goodall, Menzies, et al., 2020), we also hypothesised that the training‐induced changes in sprint cycling torque measures and cycling PPO would be related.

## 2 MATERIALS AND METHODS

### 2.1 Participants

Fourteen recreationally active (as defined by McKay et al., 2022) individuals (mean ± SD; *n* = 8 males, age 28 ± 5 years, height 181.6 ± 9.6 cm, body mass 83.9 ± 13.2 kg, 3RM back squat 113 ± 17 kg, relative 3RM back squat 1.4 ± 0.4 kg.kg^−1^; *n* = 6 females, age 30 ± 8 years, height 170.0 ± 4.8 cm, body mass 67.3 ± 3.9 kg, 3RM back squat 74 ± 15 kg, relative 3RM back squat 1.1 ± 0.2 kg.kg^−1^) who were strength‐trained (group strength training experience; 2.3 ± 1.6 years, group habitual training 5.3 ± 2.6 h.wk^−1^) and novice cyclists, volunteered for this study. Participants were required to have more than six months of strength‐training experience and should be completing a minimum of two 1‐h strength sessions per week to be included. Participants were also required to have completed an average of less than 2‐h cycling per week for the past 12 months. A medical questionnaire confirmed that participants had no adverse cardiovascular or musculoskeletal risk factors. Before the commencement of the study, participants provided written informed consent. Ethics approval was provided by the host institution's Ethics Committee.

### 2.2 Experimental overview

A single‐group, longitudinal (pre‐test/post‐test) study design was employed to determine the effects of a 6‐week training program consisting of sprint cycling (Table S1) and resistance training (Figure S1). Prior to (pre‐testing; 6 ± 3 days) and following (post‐testing; 3 ± 1 day) the 6‐week training programme, participants completed an IMTP and sprint cycling testing protocol (described below), with each protocol separated by 30‐min passive rest. All participants performed a familiarisation session within seven days of pre‐testing, where each protocol was performed in a randomised order and anthropometric data (height and body mass) were collected. Protocol order was kept consistent from pre‐to post‐training testing for each participant. Participants body mass was also collected on arrival at pre‐ and post‐testing. Pre‐ and post‐testing sessions were completed at the same time of the day (±1 h) to avoid diurnal fluctuations in performance (Teo et al., 2011). For all testing sessions, participants were requested to refrain from ingesting stimulants or depressants for 12 h, strenuous exercise for 36 h, and to arrive 3 h post‐prandial in a well‐hydrated state. Participants were requested to refrain from performing any additional resistance‐type or high‐intensity training for the duration of the study (confirmed verbally post‐study).

### 2.3 6‐Week training programme

Within each week of the programme, participants were prescribed three ergometer sprint cycle sessions (Table S1) and three gym‐based resistance training sessions (Figure S1). The aims of the resistance training program were to develop lower and upper body maximal strength and RFD capabilities. The aims of the cycling program were to develop all areas of the cycling power‐ and torque‐cadence relationships. All training sessions were supervised by the lead researcher.

Training program sprint cycle sessions commenced with a 15‐min standardised warm‐up. Training program sprint cycle sessions were performed on an ergometer (Wattbike Ltd), fitted with clipless pedals. Participants were fitted with cycling shoes (Shimano RP1, Sakai City, Japan) and instructed to “attack the effort as fast and as hard as possible” for all sprints, with strong verbal encouragement provided throughout (Kordi, Folland, Goodall, Menzies, et al., 2020).

Participants commenced training program gym sessions with a warm‐up and plyometrics (Figure S1). Participants were prescribed a bilateral, compound, multi‐joint key lift (back squat and trap bar deadlift), a secondary key exercise (hip thrusts and dynamic mid‐thigh clean pull), a unilateral exercise (box step up, rear foot elevated split squat and barbell lunge press), as well as supplementary exercises. The key lifts consisted of 3 sets of 5 repetitions, with a 2–3‐s descent, a maximal mobilisation of the load in the concentric phase, and 3–5‐min recovery between sets. Participants were instructed to perform the concentric phase of all key lifts “as fast and as hard as possible”. To determine dynamic lower‐body strength, back squat 3RM was assessed during resistance session one on weeks 1 and 6 of the training programme. Trap bar deadlift 3RM was assessed during resistance session two on weeks 1 and 6 of the training program. Each 3RM testing session was preceded by at least 36‐h rest and replicated the protocol of Darrall‐Jones et al. (2015). The 3RM data on week one were used for loading prescriptions for key lifts for the remainder of the program (Munro & Haff, 2018).

### 2.4 Isometric Mid‐Thigh Pull (IMTP)

Before the maximal IMTP testing, participants performed a standardised warm‐up (Guppy et al., 2022). Following 2 min of passive rest, participants were placed in a posture and bar position corresponding to the start of the second pull of the power clean (Haff et al., 1997) with hip and knee angles of 146 ± 4° and 142 ± 3°, respectively. Participants then performed one set of five 1‐s and one set of five 5‐s IMTP trials, with a 1‐min passive rest between trials and a 10‐min passive rest between sets. Set order was randomised during the familiarisation session and then standardised throughout. Participants were instructed to complete each trial “as fast and as hard as possible” for the 1‐s trials, and as “hard and as fast as possible” for the 5‐s trials (Guppy et al., 2022). The equipment used, standardisation of the set‐up (i.e., joint angles, bar height, hand grip width, foot position), individual trial countdown, implementation of the pull and in‐session trial exclusion criteria replicated the methods described previously by Guppy et al. (2022).

No filtering was applied to the force‐time data during analysis (Dos’Santos et al., 2018). All collected force‐time curves were analyzed using custom software (LabVIEW, Version 14.0, National Instruments). Force onset was defined as “the last peak/trough before the signal deflects away from baseline noise” (Tillin et al., 2010) and identified manually using previously outlined methods (Guppy et al., 2021). Peak force (PF) was defined as the maximum force in each trial minus the participants' body mass. Peak RFD (RFD_peak_) was the fastest RFD during any 20‐ms sampling window (Haff et al., 2015). Early and late RFD (or RTD for the cycling protocol below) were defined as RFD in the time bands 0–100 ms (RFD_0‐100_) and 0–200 ms (RFD_0‐200_), respectively, with both calculated as the quotient of the changes in force and time. Average RFD (RFD_avg_) was calculated as the change in force from force onset to PF divided by the time elapsed (Haff et al., 2015). In accordance with previous recommendations, data for all RFD variables were derived from the 1‐s IMTP, while PF was derived from the 5‐s IMTP (Guppy et al., 2022). Once processed, the means of the 3 “best trials” within each set in each testing session were used for statistical analysis. The definitions for “best trials” replicated Connolly et al. (2023).

### 2.5 Sprint cycle protocol (pre‐ and post‐programme testing)

Cycling performance tests were performed on a Velotron cycle ergometer (Dynaﬁt Pro Velotron; RacerMate), which was fitted with clipless pedals. Ergometer dimensions were adjusted to a comfortable position for each participant during familiarisation and were standardised throughout. Participants wore cycling shoes (Shimano RP1) fitted with cleats. The ergometer was fitted with 172.5 mm Infocrank powermeter cranks (Verve Cycling, Perth, Australia) that measured left and right crank torques independently. Once‐per‐revolution power, cadence and torque measurements (256 Hz analogue‐digital conversion rate) were recorded via customised Infocrank data logger software (Infocrank, Australia) and stored on a mobile phone (Sony Experia Z3 Compact). The warm‐up and main set were controlled by Velotron Coaching and Wingate software (RacerMate Inc), respectively.

The sprint cycle protocol commenced with participants performing a standardised 15‐min warm‐up. After 5‐min passive rest, participants performed three 5‐s sprints initiated from stationary starts against external resistances of 0.2, 0.4, and 0.6 Nm.kg^−1^ and two 5‐s sprints initiated from rolling starts (20‐s lead in) with an initial cadence of ∼80 rpm and external resistances of 0.0 and 0.2 Nm.kg^−1^. All sprints were separated by 5 min of passive rest. Sprints were conducted in a randomised order during familiarisation and were standardised throughout. Vigorous verbal encouragement was provided throughout each sprint, where participants were requested to remain seated and keep their hands on the dropped portion of the handlebars. For all stationary start sprints, the crank starting position of the lead sprint leg was standardised at 90° (0° = Top dead center) using a wooden block as this position was easiest to standardise. The lead sprint leg was self‐selected by the participants during familiarisation and was standardised throughout.

All collected torque, power and cadence data were downloaded and processed using Microsoft Excel 2010 (Microsoft Excel, 2010). All RTD measurements were calculated using the average of downstrokes 2 and 3 from the 0.6 Nm.kg^−1^ sprint replicating the methods of Connolly et al. (2022). The definition for torque onset and the calculation of RTD_0‐100_, RTD_0‐200,_ RTD_avg_ and RTD_peak_ mirrored that which was used in the IMTP protocol. Once individual downstrokes were processed, RTD measures were averaged for downstrokes 2 and 3.

The maximum torque produced within the subset of all downstrokes in all sprints in each testing session (i.e., pre‐ and post‐testing) was recorded as the peak torque (*T*_peak_). The observed (i.e., actual) peak cadence (RPM_peak_) and PPO were the highest observed cadence and power in all sprints in each testing sessions (Table 1). In addition, power‐cadence (P‐C) and torque‐cadence (T‐C) relationships were developed using the mean torque values for each pedal stroke and the cadence and power data using the processing methods described by Connolly et al. (2023). The T‐C and P‐C relationships were established by fitting linear and third‐order polynomial regressions, respectively (Connolly et al., 2023; Wackwitz et al., 2021). The *y*‐intercept was set at zero for the P‐C relationship. The apex of the P‐C relationship was interpolated to derive theoretical peak power (*P*_max_) and cadence at *P*_max_ (i.e., optimal cadence; RPM_opt_). Theoretical maximal torque (*T*_0_) and maximal cadence (RPM_max_) were the extrapolated *y*‐ and *x*‐intercepts of the torque‐cadence relationship. Individual P‐C and T‐C relationships were modelled from 19.5 ± 2.5 (mean ± SD) data points and had an *r* ^*2*^ equal to 0.997 ± 0.003 and 0.983 ± 0.014, respectively.

**TABLE 1:** Pre‐ and post‐sprint cycling, isometric mid‐thigh pull (IMTP) and three repetition maximum (3RM) testing values in strength‐trained novice cyclists.

Protocol	Measure	Pre (Week 1 for 3RM)	Post (Week 6 for 3RM)	% change	*p*‐value	Hedge's effect size (95%CI)
**Cycling**	PPO (W)	1071 ± 250	1186 ± 246	10.7	<0.01	0.45 (−0.30–1.20)
	PPO:BM (W/kg)	13.9 ± 2.3	15.4 ± 2.0	10.5	<0.01	0.68 (−0.09–1.44)
	RPM_peak_ (rpm)	185 ± 15	192 ± 14	4.3	<0.01	0.47 (−0.28–1.22)
	T_peak_ (Nm)	207.1 ± 37.8	231.4 ± 35.1	11.7	<0.01	0.65 (−0.11–1.41)
	RTD_0‐100_ (Nm.s^−1^)	291.2 ± 111.4	456.4 ± 171.9	56.7	<0.01	1.11 (0.31–1.90)
	RTD_0‐200_ (Nm.s^−1^)	522.9 ± 176.9	716.1 ± 224.7	36.9	<0.01	0.93 (0.15–1.71)
	RTD_avg_ (Nm.s^−1^)	507.0 ± 151.9	657.5 ± 186.3	29.7	<0.01	0.86 (0.09–1.63)
	RTD_peak_ (Nm.s^−1^)	864.9 ± 252.0	1106.2 ± 296.2	27.9	<0.01	0.85 (0.08–1.63)
	*T*_0_ (Nm)	165.2 ± 25.0	182.0 ± 29.2	10.2	<0.01	0.60 (−0.16–1.36)
	*P*_max_ (W)	1066.6 ± 255.4	1171.9 ± 265.7	9.9	<0.01	0.39 (−0.36–1.14)
	RPM_max_ (rpm)	232.2 ± 19.4	234.1 ± 19.1	0.8	0.44	0.10 (−0.65–0.84)
	RPM_opt_ (rpm)	118 ± 10	119 ± 9	0.8	0.77	0.10 (−0.64–0.84)
**IMTP**	Peak force (N)	1811.7 ± 573.6	2049.8 ± 608.4	13.1	<0.01	0.39 (−0.36–1.14)
	RFD_0‐100_ (N.s^−1^)	3303.4 ± 1862.0	4246.6 ± 1828.5	28.6	<0.01	0.50 (−0.26–1.25)
	RFD_0‐200_ (N.s^−1^)	2786.7 ± 1146.9	3520.1 ± 1179.2	26.3	<0.01	0.61 (−0.15–1.37)
	RFD_avg_ (N.s^−1^)	2852.5 ± 1465.4	3778.8 ± 2364.0	32.5	0.02	0.46 (−0.29–1.21)
	RFD_peak_ (N.s^−1^)	11,344.2 ± 5608.7	14,030.1 ± 6419.9	23.7	<0.01	0.35 (−0.40–1.09)
**3RM**	Back squat (kg)	96 ± 25	108 ± 25	12.5	<0.01	0.47 (−0.28–1.22)
	Trap bar deadlift (kg)	106 ± 35	119 ± 36	12.3	<0.01	0.36 (−0.39–1.10)

*Note*: Pre‐ and post‐values expressed as mean ± SD (*n* = 14). Hedge's effect size presented with 95% confidence intervals (CI).

Abbreviations: *P*_max_, theoretical peak power; PPO, observed peak power output; PPO:BM, PPO relative to body mass; RFD/RTD_0‐100_ and RFD/RTD_0‐200_, rate of force/torque development from 0 to 100 ms and 0–200 ms; RFD/RTD_avg_, average rate of force/torque development; RFD/RTD_peak_, peak rate of force/torque development; RPM_max_, theoretical peak cadence; RPM_opt_, theoretical optimal cadence; RPM_peak_, observed peak cadence; *T*_0_, theoretical peak torque; T_peak_, observed peak torque.

Statistical significance set at *p* < 0.05.

### 2.6 Statistical analysis

**FIGURE 1:**
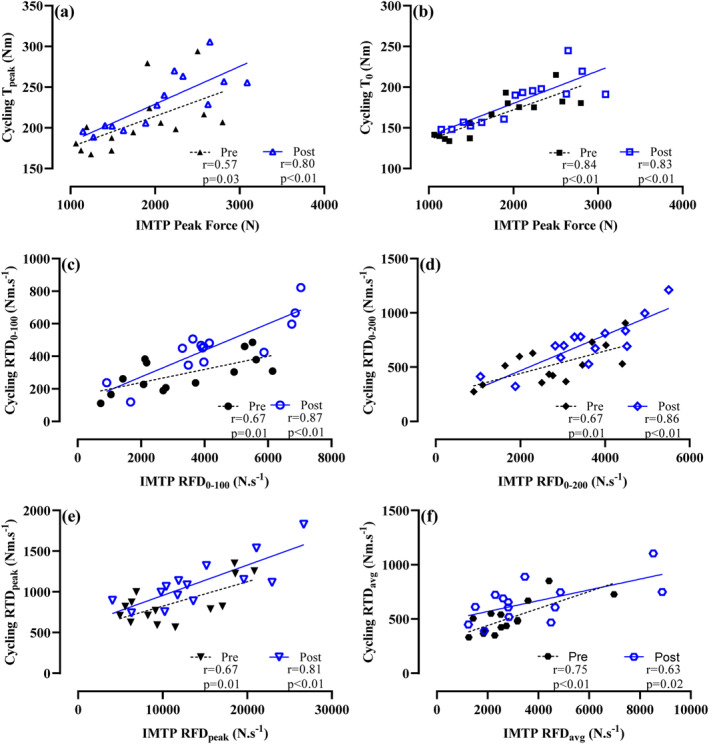
Pearson's correlation coefficients (*r*) between isometric mid‐thigh pull (IMTP) force and sprint cycling torque measures pre‐ and post‐training program. (A) Observed cycling peak torque (T_peak_) and IMTP peak force, (B) theoretical cycling peak torque (*T*_0_) and IMTP peak force, (C) rate of force/torque development (RFD/RTD) from 0 to 100 ms (RFD/RTD_0‐100_), (D) RFD/RTD from 0 to 200 ms (RFD/RTD_0‐200_), (E) peak RFD/RTD (RFD/RTD_peak_) and (F) average RFD/RTD (RTD_avg_).

**FIGURE 2:**
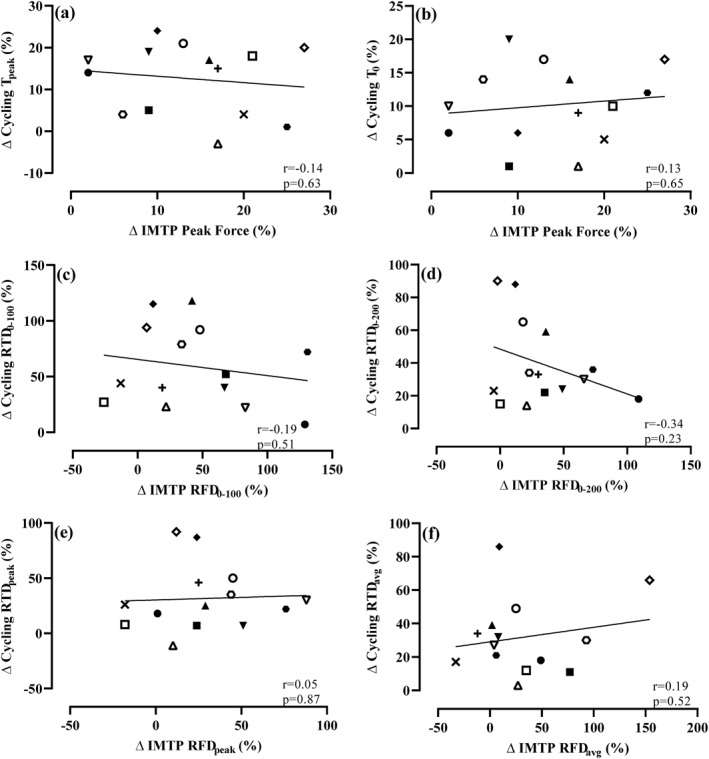
Pearson's correlation coefficients (*r*) between the relative change (Δ) in cycling torque measures and relative change in isometric mid‐thigh pull (IMTP) force measures pre‐to post‐training. (A) Observed cycling peak torque (T_peak_) and IMTP peak force, (B) theoretical cycling peak torque (*T*_0_) and IMTP peak force, (C) rate of force/torque development (RFD/RTD) from 0 to 100 ms (RFD/RTD_0‐100_), (D) RFD/RTD from 0 to 200 ms (RFD/RTD_0‐200_), (E) peak RFD/RTD (RFD/RTD_peak_) and (F) average RFD/RTD (RTD_avg_). Each participant is represented by a specific symbol shape in graphs a‐f.

Descriptive statistics are reported as mean ± SD (Table 1). A linear mixed‐effect model using the R package (version 4.0.2, R Core Team, 2020) lme4 (Bates et al., 2015) was used to determine whether differences existed between pre‐ and post‐test IMTP, sprint cycling and 3RM variables. Accordingly, fixed effects were gender and timepoint, whereas participants were a random effect. Pairwise comparisons were performed using R emmeans package (Lenth, 2020). Visual inspection of the residual plots using R easystats performance package (Lüdecke et al., 2021) confirmed that linear modelling assumptions were met. Statistical significance was accepted at *p* < 0.05. Hedge's effect sizes (g) were calculated (with 95% confidence intervals) in a custom script (Hedges & Olkin, 1985) with bias corrected (Table 1) to estimate the magnitude of performance change and interpreted as trivial (<0.2), small (0.21–0.60), moderate (0.61–1.20), large (1.21–2.0), very large (2.1–4.0) and extremely large (>4.0) (Hopkins et al., 2009). Pearson's product–moment correlations (r) were computed in Prism GraphPad (Version 9.2.0) and used to examine the relationship between the following: (i) IMTP force and sprint cycling torque pre‐ and post‐training (Figure 1); (ii) the training‐induced change in IMTP force and cycling torque (Figure 2); and (iii) the training‐induced change in cycling PPO and *P*_max_ with cycling torque, IMTP force and 3RM (Table 2). The Hopkins‐modified Cohen's scale was used to describe the relationships as follows: <0.1, trivial; 0.1–0.3, small (weak); 0.3–0.5, moderate; 0.5–0.7, large (strong); 0.7–0.9, very large (very strong); and >0.9, almost perfect (Hopkins et al., 2009).

**TABLE 2:** Relationships between training‐induced changes in cycle power output and various test measures in strength‐trained novice cyclists.

Predictor variable	PPO		*P*_max_	
	*p*	*r*	*p*	*r*
Cycling T_peak_ (Nm)	0.38	0.25	0.34	0.27
Cycling RPM_peak_ (rpm)	0.27	0.32	0.11	0.44
Cycling *T*_0_ (Nm)	0.10	0.46	0.07	0.50
Cycling RTD_0‐100_ (Nm.s^−1^)	0.59	0.16	0.45	0.22
Cycling RTD_0‐200_ (Nm.s^−1^)	0.84	0.06	0.58	0.16
Cycling RTD_avg_ (Nm.s^−1^)	0.88	−0.05	0.87	0.05
Cycling RTD_peak_ (Nm.s^−1^)	0.94	−0.02	0.99	−0.001
IMTP peak force (N)	0.43	0.23	0.48	0.21
IMTP RFD_0‐100_ (N.s^−1^)	0.59	−0.16	0.62	−0.15
IMTP RFD_0‐200_ (N.s^−1^)	0.33	−0.28	0.30	−0.30
IMTP RFD_avg_ (N.s^−1^)	0.74	0.10	0.82	0.07
IMTP RFD_peak_ (N.s^−1^)	0.92	0.03	0.77	0.09
3RM back squat (kg)	0.84	−0.06	0.56	−0.17
3RM trap bar deadlift (kg)	0.38	−0.25	0.37	−0.26

*Note*: *r*, Pearson's correlation coefficients

Abbreviations: *P*_max_, cycling theoretical peak power; PPO, cycling observed peak power output; RFD/RTD_0‐100_ and RFD/RTD_0‐200_, rate of force/torque development from 0 to 100 ms and 0–200 ms; RFD/RTD_avg_, average rate of force/torque development; RFD/RTD_peak_, peak rate of force/torque development; RPM_peak_, cycling observed peak cadence; *T*_0_, cycling theoretical peak torque; T_peak_, cycling observed peak torque.

Statistical significance set at *p* < 0.05.

## 3 RESULTS

Training resulted in significant improvements in all cycling power and torque measures, cycling RPM_peak_, all IMTP force measures, and both 3RM measures in week 6 of the training program (*p* < 0.05; Table 1). No significant differences were observed in cycling RPM_max_ and RPM_opt_ following training (*p* > 0.05; Table 1).

Strong‐to‐very strong positive relationships were observed between IMTP forces and cycling torque measures both pre‐ and post‐training (Figure 1). No significant relationships were observed between the training‐induced changes in IMTP force measures and sprint cycling torque measures following training (Figure 2). No significant relationships were observed between the training‐induced change in PPO or *P*_max_ and the change in cycling torque, IMTP force and 3RM (Table 2).

## 4 DISCUSSION

The aims of the present study were to investigate: (i) the extent that sprint cycling torque and IMTP force measures change following training; and (ii) the relationship between the training‐induced changes in sprint cycling torque, cycling power (PPO/*P*_max_), IMTP force and 3RM measures. The following findings were observed: (1) significant training‐induced increases in cycling PPO (10.7%), *T*_0_ (10.2%), RTD (27.9%–56.7%), IMTP PF (13.1%) and RFD (23.7%–32.5%); (2) no significant relationships between the training‐induced changes in IMTP force and sprint cycling torque; and (3) no significant relationships between the training‐induced changes in cycling PPO or *P*_max_ and changes in IMTP force, cycling torque and 3RM measures. The divergent training‐induced changes in these assessments indicate that these tests are affected by different underpinning mechanisms, likely providing information on different aspects of neuromuscular function when measured following a period of training.

The present study indicates that as little as 6 weeks of resistance and sprint cycling training results in substantial improvements in cycling performance in strength‐trained novice cyclists. Indeed, the observed 10.7% increase in cycling PPO in the present study is greater than the 6.7% increase achieved following a 3‐month talent development sprint cycling program with developmental cyclists (Tofari et al., 2017), or the 3.0% increase following 6‐week of cycling specific isometric‐training stimulus in world‐class sprint cyclists (Kordi, Folland, Goodall, Menzies, et al., 2020). The steep improvement curve in cycling measures in the present study is not unexpected given that the participants were starting from a low experience base in cycling. The improvements in the present study (Table 1) align with previous research reporting improvements in RFD/RTD in as little as 2–6 weeks training, as well as research reporting the torque component of PPO to be more trainable than the cadence component (Dorel, 2018; Douglas et al., 2021; Kordi, Folland, Goodall, Menzies, et al., 2020). Training‐induced changes in torque may be attributable to neural adaptations, such as increased motor unit recruitment, rate of coding and synchronisation; or peripheral adaptations, such as increased muscle cross‐sectional area or increased pennation angle (Andersen & Aagaard, 2006; Andersen et al., 2010; Douglas et al., 2021; Kordi, Folland, Goodall, Haralabidis, et al., 2020; Maffiuletti et al., 2016). The present data indicate that, in the current population, an emphasis should be placed on systematically developing strength as substantial increases in torque (and PPO/Pmax) can be achieved in a relatively short period of time. However, it is noteworthy that our study design also included low torque/high cadence efforts with the goal of improving limits to the power–cadence relationship. As such it is plausible that such high cadence training also contributed to the improvements in power observed and should also be considered within a structured training program (Dorel, 2018; Douglas et al., 2021).

As hypothesised, we did not observe a relationship between the training‐induced changes in IMTP force and sprint cycling torque measures. In the present study, group percentage increases were greater in IMTP PF (13.1%) compared to sprint cycling Tpeak (11.7%) and T0 (10.2%), while most cycling RTD measures increased by a greater percentage (27.9%–56.7%) compared to IMTP RFD (23.7%–32.5%). The greater group percentage increases in IMTP compared to cycling peak values may be surprising given the participants had more experience in strength training than cycling. However, only 1 participant had previous experience at completing the IMTP, so the groups were relatively novice in this exercise, and improvement may be expected after 6 weeks of targeted training. The divergent training‐induced changes in the present study are likely due to differences in the underpinning mechanisms associated with the production of IMTP force and sprint cycling torque. Indeed, our earlier work (Connolly et al., 2023) showed a stronger relationship between peripheral neuromuscular function mechanisms and cycling torque and between central neuromuscular function mechanisms and IMTP force. Other reasons for divergent training‐induced changes in IMTP force and sprint cycling torque include differences in the muscle activation strategies and motor unit recruitment in isometric and dynamic actions (Murphy & Wilson, 1996), or the absence of the stretch‐shortening cycle in isometric actions (in contrast to dynamic action) (Wilson et al., 1991), in addition to musculoskeletal stiffness providing a greater contribution to force production within isometric compared to dynamic tasks (James et al., 2023; Wilson et al., 1994). The results of the present study indicate that while there is a level of transferability in an individual's ability to rapidly produce IMTP force and sprint cycling torque at a single timepoint, these measures change at a different rate in response to training in strength‐trained novice cyclists. From an applied perspective, these results emphasise why it is important that cross‐sectional relationships are not assumed to hold true when examined longitudinally following training (James et al., 2023).

Findings from the present study contrast our second hypothesis of a relationship between the training‐induced changes in cycling *P*_max_, cycling torque, IMTP force or 3RM measures. While previous research (Dorel, 2018; Dorel et al., 2005; Douglas et al., 2021; Kordi et al., 2017) highlights the importance of strength capacities for sprint cycling power production, our data show that the strength measures assessed here (cycling torque, IMTP force, 3RM) do not change at the same rate as cycling PPO or *P*_max_ after a short training period in strength‐trained novice cyclists. The assessed strength measures may be useful to a practitioner interested in the progression of strength; however, our results indicate that these strength changes may not be transferable or related to cycling power changes following a short (e.g., 6 weeks) training intervention in the current population. The divergent changes in strength measures and PPO/*P*_max_ in the current study agree with a previous study in well‐trained endurance cyclists (Rønnestad et al., 2010), but contradicts findings in world‐class cyclists (Kordi, Folland, Goodall, Menzies, et al., 2020), where a moderate relationship (*r* = 0.42–0.47; *p* < 0.05) between the training‐induced changes in knee extension late RTD (150–200 ms) and cycling *P*_max_ were observed following a 6‐week isometric cycling specific intervention. The differences in study results mentioned above may be explained in part by the experience level of the cyclists and the differences in the assessments used.

As with any investigation, there are limitations to our work that should be considered. Firstly, we acknowledge that the training background of the participants in the present study will have influenced the findings, and therefore, our results are applicable specifically to individuals who are strength‐trained novice cyclists. By recruiting individuals with more experience in strength than cycling, we increased the likelihood of seeing a divergent adaptation in the strength and cycling assessments, and therefore, increased the likelihood of determining the relationship between force and torque measures (or not). Next, we acknowledge that the small sample size for the present study may have affected the relationships presented. Further research is needed to ascertain whether relationships in the present study are similar in other populations such as strength‐trained, sprint cyclists. Finally, we are aware that several measures such as cycling *P*_max_ normalised by the frontal area, or 15‐s to 30‐s cycling PPO have a strong relationship with specific event measures (such as the flying 200‐m velocity) or with event specific durations of 15–30 s (Dorel et al., 2005; Ferguson et al., 2023). However, the present study was not designed to examine the factors that may be correlated with performance. Instead, we investigated the factors that may be correlated with RFD/RTD. We acknowledge that future research should investigate the factors that may be correlated with performance.

## 5 CONCLUSION

In conclusion, training resulted in substantial increases in cycling power and torque measures in strength‐trained novice cyclists. The increase in cycling power was mediated by increases in cycling torque, IMTP force and 3RM; however, these measures did not change at the same rate. Our results indicate no relationship between the training‐induced changes in IMTP force and sprint cycling torque following six weeks of training in strength‐trained novice cyclists. These data suggest that training‐induced changes in IMTP and 3RM are not indicative of training‐induced changes in sprint cycling in this population. The divergent training‐induced changes in these assessments suggest that these tests are potentially affected by different underpinning mechanisms, likely providing information on different aspects of neuromuscular function when measured following training.

## CONFLICT OF INTEREST STATEMENT

The authors report there are no competing interests to declare.

## Supporting information

Supporting Information S1

Supporting Information S2

## Data Availability

The authors confirm that the data supporting the findings of this study are available within the article.
