# Supplementary material for: Change in sprint cycling torque is not associated with change in isometric force following six weeks of sprint cycling and resistance training in strength‐trained novice cyclists
Source: Eur J Sport Sci. 2024 Oct 16;24(11):1604–13. doi: 10.1002/ejsc.12203 (PMC11534655; doi:10.1002/ejsc.12203)
Supplement: Supplementary file 1 — Supporting Information S1 [file EJSC-24-1604-s001.pdf]

Supplementary Table 1. Summary of the generic sprint cycling training prescription. Session details below as number of sets x [repetitions x duration/rest between repetitions]/rest between sets.

Session	Description	Week 1	Week 2	Week 3	Week 4	Week 5	Week 6
1	Low Torque, low resistance	1 x [5 x 5 s/ 5 min]	1 x [4 x 8 s/ 5 min]	1 x [5 x 8 s/ 5 min]	1 x [5 x 8 s/ 5 min]	1 x [6 x 5 s/ 5 min]	1 x [4 x 5 s/ 5 min]
2	High Torque, high resistance	4 x [2 x 8 s/ 1 min] /5 min	4 x [2 x 8 s/ 1 min] /5 min	5 x [2 x 8 s/ 1 min] /5 min	5 x [2 x 8 s/ 1 min] /5 min	4 x [2 x 8 s/ 1 min] /5 min	3 x [2 x 8 s/ 1 min] /5 min
3	High Power, moderate resistance	4 x [2 x 6 s/ 1 min] /5 min	5 x [2 x 6 s/ 1 min] /5 min	6 x [2 x 6 s/ 1 min] /5 min	6 x [2 x 6 s/ 1 min] /5 min	5 x [2 x 6 s/ 1 min] /5 min	3 x [2 x 6 s/ 1 min] /5 min
