# Supplementary material for: Change in sprint cycling torque is not associated with change in isometric force following six weeks of sprint cycling and resistance training in strength‐trained novice cyclists
Source: Eur J Sport Sci. 2024 Oct 16;24(11):1604–13. doi: 10.1002/ejsc.12203 (PMC11534655; doi:10.1002/ejsc.12203)
Supplement: Supplementary file 2 — Supporting Information S2 [file EJSC-24-1604-s002.pdf]

**Supplementary Figure 1.** Summary of the generic resistance training prescription. *=repetitions on each side

| Session | Exercise | | Week 1 | Week 2 | Week 3 | Week 4 | Week 5 | Week 6 |
|---|---|---|---|---|---|---|---|---|
| 1 | Back squat | Sets | 3RM testing | 3 | 3 | 3 | 3 | 3RM testing |
| | | Reps | | 5, 5, 5 | 5, 5, 5 | 5, 5, 5 | 5, 5, 5 | |
| | | % 3RM | | 70, 75, 80 | 75, 80, 85 | 80, 85, 90 | 72.5, 77.5, 82.5 | |
| | Box step up | Sets | 3 | 3 | 3 | 3 | 3 | 3 |
| | | Reps* | 5, 5, 5 | 5, 5, 5 | 5, 5, 5 | 5, 5, 5 | 5, 5, 5 | 5, 5, 5 |
| | Barbell pendlay row | Sets | 3 | 3 | 3 | 3 | 3 | 3 |
| | | Reps | 5, 5, 5 | 5, 5, 5 | 5, 5, 5 | 5, 5, 5 | 5, 5, 5 | 5, 5, 5 |
| | Single-leg RDL | Sets | 3 | 3 | 3 | 3 | 3 | 3 |
| | | Reps* | 5, 5, 5 | 5, 5, 5 | 5, 5, 5 | 5, 5, 5 | 5, 5, 5 | 5, 5, 5 |
| | Calf raises (straight leg) | Sets | 3 | 3 | 3 | 3 | 3 | 3 |
| | | Reps* | 5, 5, 5 | 5, 5, 5 | 5, 5, 5 | 5, 5, 5 | 5, 5, 5 | 5, 5, 5 |
| | Back extensions | Sets | 3 | 3 | 3 | 3 | 3 | 3 |
| | | Reps | 15, 15, 15 | 15, 15, 15 | 18, 18, 18 | 18, 18, 18 | 20, 20, 20 | 20, 20, 20 |
| | Glute bridge | Sets | 3 | 3 | 3 | 3 | 3 | 3 |
| | | Reps | 15, 15, 15 | 15, 15, 15 | 18, 18, 18 | 18, 18, 18 | 20, 20, 20 | 20, 20, 20 |
| 2 | Trap bar deadlift | Sets | 3RM testing | 3 | 3 | 3 | 3 | 3RM testing |
| | | Reps | | 5, 5, 5 | 5, 5, 5 | 5, 5, 5 | 5, 5, 5 | |
| | | % 3RM | | 70, 75, 80 | 75, 80, 85 | 80, 85, 90 | 72.5, 77.5, 82.5 | |
| | Hip thrusts | Sets | 3 | 3 | 3 | 3 | 3 | 3 |
| | | Reps | 5, 5, 5 | 5, 5, 5 | 5, 5, 5 | 5, 5, 5 | 5, 5, 5 | 5, 5, 5 |
| | Rear foot elevated split squat | Sets | 3 | 3 | 3 | 3 | 3 | 3 |
| | | Reps* | 5, 5, 5 | 5, 5, 5 | 5, 5, 5 | 5, 5, 5 | 5, 5, 5 | 5, 5, 5 |
| | Barbell bench press | Sets | 3 | 3 | 3 | 3 | 3 | 3 |
| | | Reps | 5, 5, 5 | 5, 5, 5 | 5, 5, 5 | 5, 5, 5 | 5, 5, 5 | 5, 5, 5 |
| | Quad superman | Sets | 3 | 3 | 3 | 3 | 3 | 3 |
| | | Reps | 15, 15, 15 | 15, 15, 15 | 18, 18, 18 | 18, 18, 18 | 20, 20, 20 | 20, 20, 20 |
| | Russian twists | Sets | 3 | 3 | 3 | 3 | 3 | 3 |
| | | Reps | 50, 50, 50 | 50, 50, 50 | 60, 60, 60 | 60, 60, 60 | 70, 70, 70 | 70, 70, 71 |
| 3 | Back squat | Sets | 3 | 3 | 3 | 3 | 3 | 3 |
| | | Reps | 5, 5, 5 | 5, 5, 5 | 5, 5, 5 | 5, 5, 5 | 5, 5, 5 | 5, 5, 5 |
| | | % 3RM | 60, 65, 70 | 60, 65, 70 | 65, 70, 75 | 70, 75, 80 | 62, 66, 70 | 62, 66, 70 |
| | Dynamic clean pull (from mid-thigh) | Sets | 3 | 3 | 3 | 3 | 3 | 3 |
| | | Reps | 5, 5, 5 | 5, 5, 5 | 5, 5, 5 | 5, 5, 5 | 5, 5, 5 | 5, 5, 5 |
| | Barbell lunge press | Sets | 3 | 3 | 3 | 3 | 3 | 3 |
| | | Reps* | 5, 5, 5 | 5, 5, 5 | 5, 5, 5 | 5, 5, 5 | 5, 5, 5 | 5, 5, 5 |
| | Pull-ups | Sets | 3 | 3 | 3 | 3 | 3 | 3 |
| | | Reps | 5, 5, 5 | 5, 5, 5 | 5, 5, 5 | 5, 5, 5 | 5, 5, 5 | 5, 5, 5 |
| | Calf raises (with bent knee) | Sets | 3 | 3 | 3 | 3 | 3 | 3 |
| | | Reps* | 5, 5, 5 | 5, 5, 5 | 5, 5, 5 | 5, 5, 5 | 5, 5, 5 | 5, 5, 5 |
| | Back hyperextension | Sets | 3 | 3 | 3 | 3 | 3 | 3 |
| | | Reps | 15, 15, 15 | 15, 15, 16 | 18, 18, 18 | 18, 18, 18 | 20, 20, 20 | 20, 20, 20 |
| | Medicine ball slams | Sets | 3 | 3 | 3 | 3 | 3 | 3 |
| | | Reps | 40, 40, 40 | 40, 40, 40 | 50, 50, 50 | 50, 50 , 50 | 60, 60, 60 | 60, 60, 60 |

**Warm up:**
2-min skipping; world's greatest stretch (8 each side); 2 sets x 10 reps body weight squats; 5 reps Inchworms; 2 sets of 10 reps walking lunges; banded Lateral shuffles (20 each side); 10 Push Ups; Shoulder taps (5 each side).

**Plyometrics:**
Session 1, Body weight vertical jump with arms; Session 2, Split squat jump; Session 3, Jump squat with 20kg barbell
(Weeks 1-4, 2 sets x 4 reps; Weeks 5-6, 3 sets x 4 reps)
